# Narrow Internal Auditory Canal With Duplication in a Patient With Sensorineural Hearing Loss and Facial Palsy: A Case Report

**DOI:** 10.7759/cureus.68957

**Published:** 2024-09-08

**Authors:** Anusha Shree Thaneeru, Rahul Suresh, Jeevika Ujjappa

**Affiliations:** 1 Radiodiagnosis, Jagadguru Jayadeva Murugarajendra Medical College, Davangere, IND; 2 Radiology, Jagadguru Jayadeva Murugarajendra Medical College, Davangere, IND

**Keywords:** duplicated iac, facial nerve, internal auditory canal, sensorineural (sn) hearing loss, vestibulocochlear nerve

## Abstract

A narrow internal auditory canal with duplication is a rare congenital disorder of temporal bone. High-resolution CT of the temporal bone is a preferred modality for assessing the bony framework of the temporal bone and inner and middle ear structures and is thus commonly used. Of late, due to rapid advancement in technology, MRI can assess the neural structures, including the vestibulocochlear and facial nerves. Here, we present the case of a 29-year-old male with congenital left-sided sensorineural hearing loss and left-sided facial palsy.

## Introduction

Among the congenital temporal bone anomalies, a narrow internal auditory canal (IAC) with duplication is relatively uncommon [1]. It is characterized by the presence of two canals, divided by a complete or incomplete bony septum. The IAC is considered to be narrow if the diameter is less than 2 mm on high-resolution CT (HRCT) of the temporal bone [2]. A narrow IAC makes up about 12% of cases of congenital temporal bone anomalies [3,4]. Often, a narrow IAC is seen in association with ipsilateral congenital sensorineural hearing loss as a consequence of aplasia or hypoplasia of the vestibulocochlear nerve or its cochlear branch, which is a relative contraindication for cochlear implantation as a mode of treatment in these patients [5,6].

The IAC is a bony canal within the petrous part of the temporal bone. Its main function is to transmit nerves and vessels from the posterior cranial fossa to the auditory and vestibular apparatus. The IAC contains the facial nerve, vestibulocochlear nerve, vestibular ganglion, and labyrinthine artery [7].

## Case presentation

A 29-year-old male presented with complaints of diminished hearing in the left ear since childhood. He gives a history of deviation of the angle of mouth toward the right, which has been progressive during his adult life. Additionally, he gives a history of inability to completely close the left eyelid since childhood. He denies any history of previous trauma, ear pain, repeated episodes of fever, ear discharge, or recurrent otitis media. He has no other known co-morbidities. There is no positive family history of hearing loss or congenital anomalies. Physical examination showed the presence of a peri-auricular skin tag.

A cranial nerve examination was performed, and targeted facial nerve assessment revealed the absence of forehead wrinkling on the left side, weak orbicularis oculi on the left, deviation of angle of the mouth toward the right, and weak puffing of the cheek on the left side, with the presence of Bell’s phenomenon on the left side, which is suggestive of Housebreakman Grade IV (moderately severe dysfunction) (Table 1).

**Table 1:** Clinical examination of the facial nerve function

Facial nerve examination	Right	Left
Wrinkling of forehead	Present	Absent
Nasolabial fold	Normal	Less prominent
Bell’s phenomenon	Absent	Present
Muscles of facial expression	Intact	Weakness present
Orbicularis oculi	Normal	Weak
Deviation of the angle of the mouth	Toward right	

Tuning fork tests were performed with lateralization toward the right on Weber’s test and Rinne’s and absolute bone conduction unappreciated on the left side (Table 2).

**Table 2:** Clinical examination for hearing: tuning fork tests

Tuning fork test	Right	Left
Rinne’s test	Positive (normal)	Cannot appreciate
Weber’s test	Lateralized to right	-
Absolute bone conduction	Not reduced (normal)	Cannot appreciate

**Figure 1:**
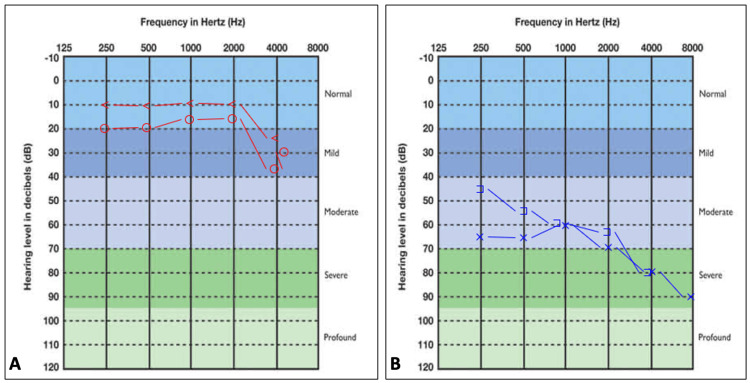
Digital pure tone audiograms of a 29-year-old male: (A) pure tone audiogram of the right ear depicting normal hearing and (B) pure tone audiogram of the left ear showing a sloping sensorineural hearing loss o - air conduction on the right side, < - bone conduction on the left side, x - air conduction on the left side, ] - masked bone conduction on the left side

The patient underwent a pure tone audiometry which revealed severe sensorineural hearing loss in the left ear with normal hearing in the right ear (Figure 1).

The patient was referred to our Department of Radiodiagnosis to rule out a possible space-occupying lesion of the brain. HRCT of the temporal bone and MRI were acquired to evaluate the seventh and eighth cranial nerves. MRI was performed using a 1.5-T MRI scanner. 3DT1 WI, T2WI, FLAIR, and 3D CISS sequences were acquired in high-resolution axial, coronal, and reformatted sagittal planes. Sagittal, para-sagittal, and coronal reformatted images of HRCT were obtained.

**Figure 2:**
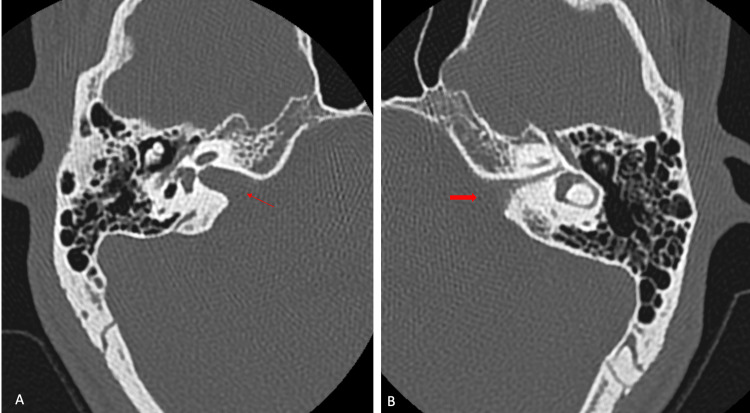
Axial planes of HRCT in bone window at level of IAC: (A) normal right IAC measuring 4.0 mm (thin red arrow) and (B) narrow left IAC measuring 1.4 mm (thick red arrow) HRCT: high-resolution CT, IAC: internal auditory canal

An HRCT of the temporal bone revealed a narrow left IAC measuring 1.4 mm in diameter with a normal right IAC measuring 4.0 mm in diameter, as shown in Figure 2.

**Figure 3:**
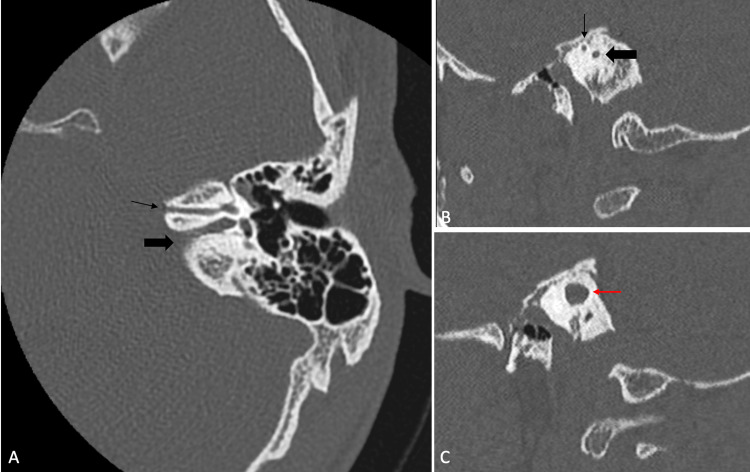
HRCT of the temporal bone in the bone window: (A) axial oblique reformatted image showing a narrow bony canal (thin black arrow) anterosuperior to the stenotic left IAC (thick black arrow), (B) sagittal reformatted images showing narrow left IAC (thick black arrow) with a thin bony canal IAC (thick black arrow) anterosuperior to it, and (3) sagittal reformatted images showing right IAC (red arrow) which is normal in diameter HRCT: high-resolution computed tomography, IAC: Internal auditory canal

An additional narrow bony canal is seen in the anterosuperior aspect of the narrow left IAC, as shown in Figure 3. Bilateral ear ossicles, cochlea, vestibule, and external auditory canal show no abnormality.

**Figure 4:**
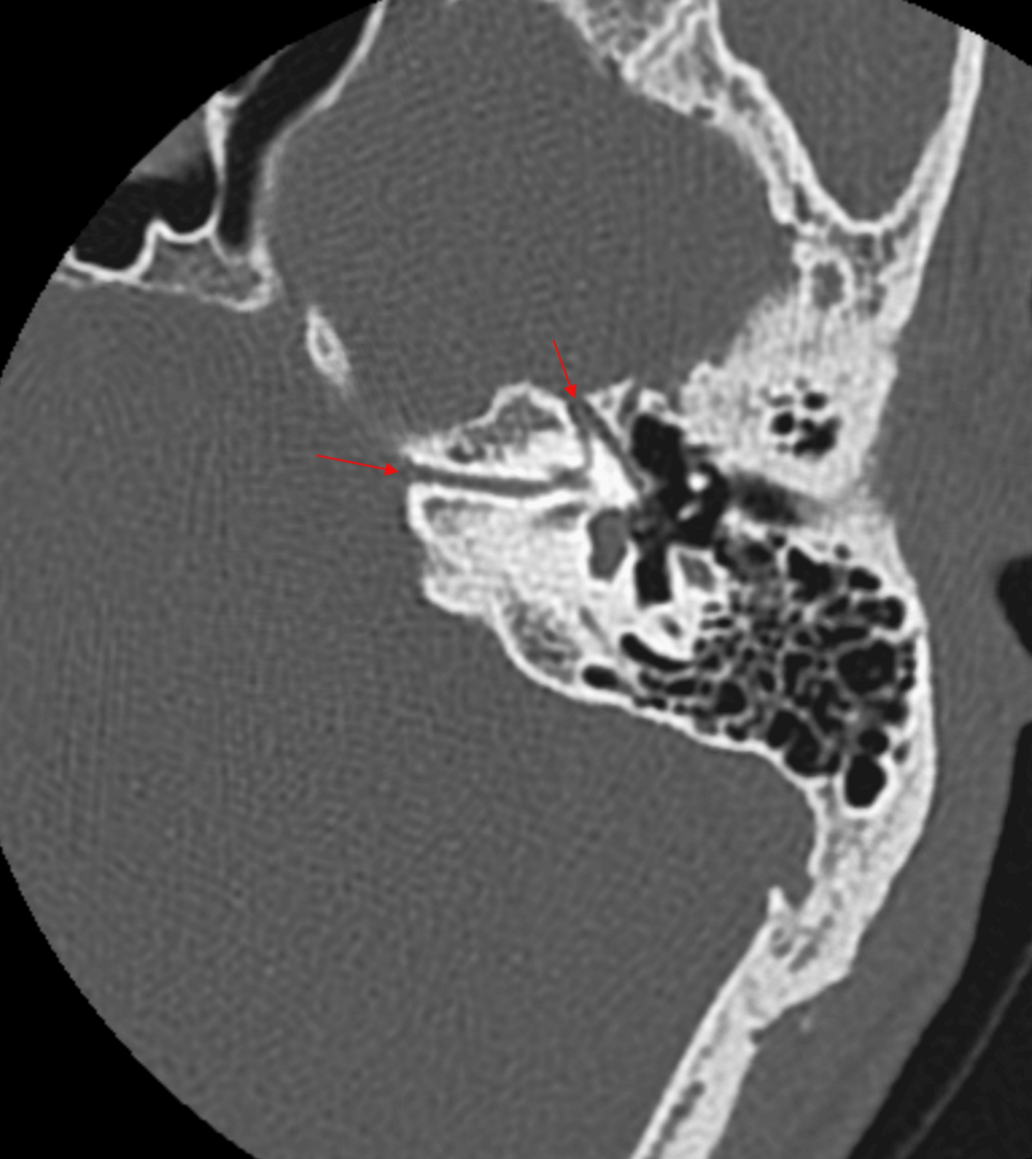
Axial oblique reformatted HRCT image in the bone window showing antero-superior bony canal in continuation with the labyrinthine segment of the left facial canal (red arrows)

The additional bony canal is seen communicating with the labyrinthine segment of the facial canal. The labyrinthine, tympanic, and mastoid segments of the left facial nerve canal appear normal (Figure 4). The right facial nerve course is normal.

**Figure 5:**
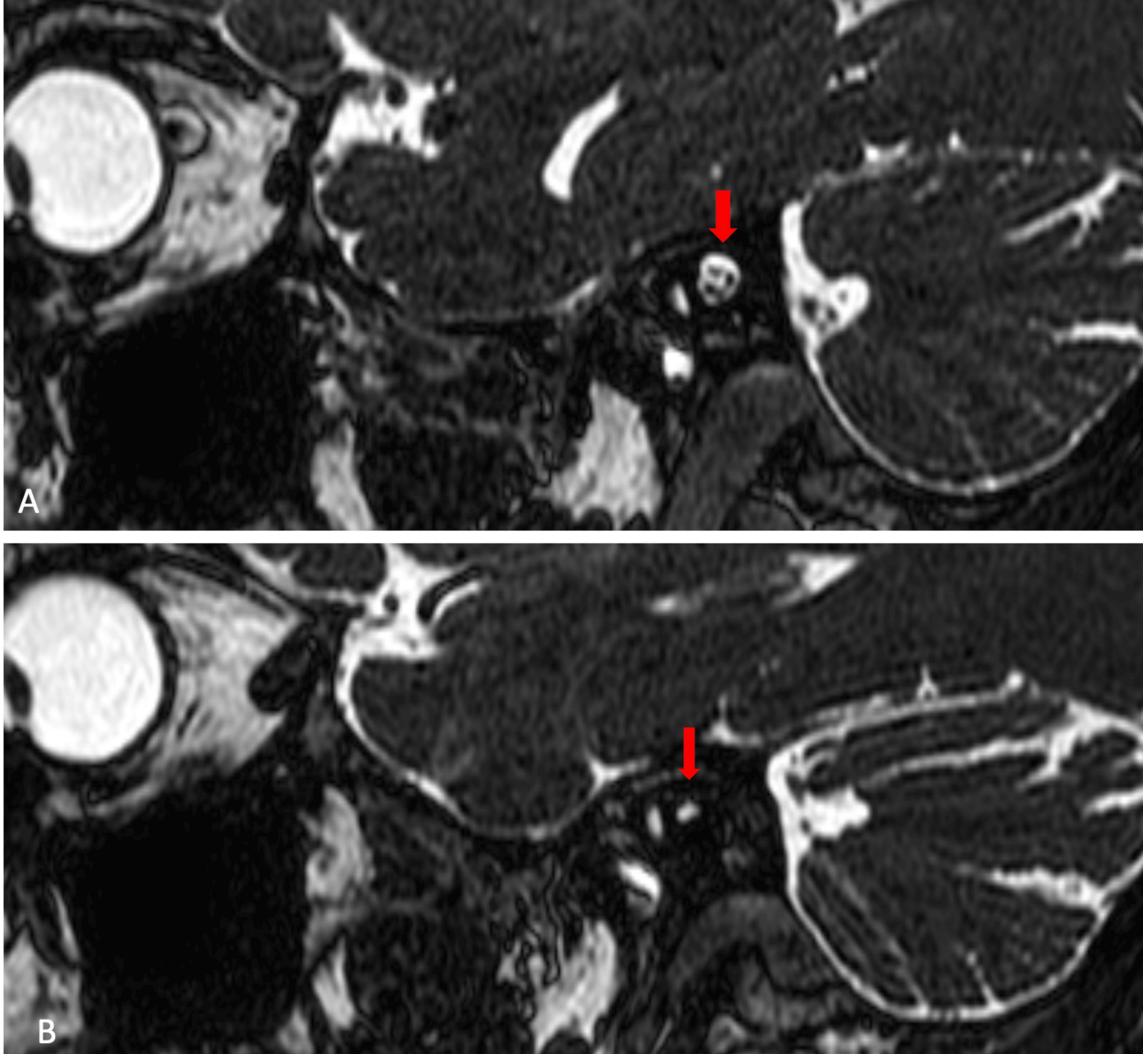
3D CISS sequence MRI in sagittal plane: (A) normal right IAC with common vestibular, cochlear, and facial nerves within and (B) hypoplastic left IAC with no identifiable neural component within 3D: three dimensional, CISS: constructive interference in steady state, MRI: magnetic resonance imaging, IAC: internal auditory canal

**Figure 6:**
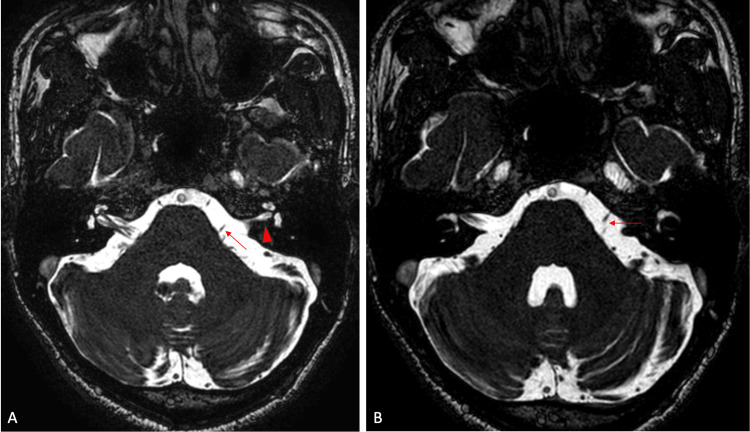
3D CISS sequence MRI images in axial plane at the level of pons: (A) hypoplastic left IAC (red arrowhead) and a hypoplastic left facial nerve arising from the lateral pons (red arrow) and (B) the hypoplastic left facial nerve coursing antero-superior to the left IAC (red arrow) 3D: three dimensional, CISS: constructive interference in steady state, MRI: magnetic resonance imaging, IAC: internal auditory canal

A 3D constructive interference in steady state (CISS) sequence was performed to evaluate cranial nerves. The right vestibulocochlear and facial nerves are normal and traversing to the right IAC. The left IAC appears hypoplastic with no identifiable neural structures within it (Figure 5). There is aplasia of the vestibulocochlear nerve. A hypoplastic facial nerve is noted arising from lateral pons coursing anterosuperior to the hypoplastic left IAC (Figure 6A-5B). The cochlea, vestibule, and semicircular canals appeared normal. The rest of the cranial nerves are normal. There is no space occupying the lesion within the neuroparenchyma.

In summary, the HRCT of the temporal bone and MRI of the brain of the 29-year-old male revealed several notable findings. There is a stenotic left IAC with an additional bony canal running anterosuperior to it. This additional canal communicates with the labyrinthine segment of the left facial canal. On the CISS sequence of the MRI, the left IAC appears hypoplastic, with no identifiable neural structures within it. Aplasia of the left vestibulocochlear nerve was observed, along with a hypoplastic facial nerve originating from the lateral pons and coursing anterosuperior to the hypoplastic left IAC.

## Discussion

The IAC is considered to be narrow if the diameter is less than 2 mm on the HRCT of the temporal bone [2]. Congenital anomalies of the inner ear associated with sensorineural hearing loss detected on CT constitute only about 20% of cases [3,4]. The normal average diameter of IAC is 4 mm [6] and is considered stenotic if the diameter is less than 2 mm [8]. Often, a narrow IAC occurs unilaterally and is associated with inner, middle, or external ear abnormalities. Systemic anomalies like polycystic kidneys, skeletal deformities, duodenal atresia, and cardiac anomalies are also commonly seen in association [9].

The embryogenesis of the inner ear and the IAC are different where the inner ear begins to develop from the 22nd day of gestation from the otic placode, and the IAC is formed by posterior ossification of mesoderm surrounding the seventh and eighth cranial nerves during the fifth and sixth month of gestation. The development of the vestibulocochlear nerve starts on the 29th day of gestation and is thought to be induced by chemotactic mechanisms of the embryonic labyrinth [1,5,9,10]. Failure in neural growth induction as a result of an abnormality in the end organs of the labyrinth is thought to be a cause of narrow IAC. It is not clear if the trophic influence of the eighth cranial nerve on the growth of IAC results in its stenosis in the case of aplasia or hypoplasia of the nerve or the primary bony defect of the IAC that inhibits the growth of the eighth cranial nerve. However, the function of the facial nerve being preserved in the majority of the cases makes the latter explanation less likely [1,5,6,9,11]. The development of the facial nerve occurs separately, which gets surrounded later as the canal develops around the eighth cranial nerve, which may cause duplication of the IAC in the case of an aplastic or hypoplastic eighth nerve.

Narrow IAC with duplication can also be seen in association with other disorders like Klippel-Feil syndrome [12] and pontine tegmental cap dysplasia [13,14]. Our case was a 29-year-old male with isolated unilateral duplicated left IAC, left vestibulocochlear nerve aplasia, and hypoplastic left facial nerve with no associated temporal bone or systemic abnormalities.

Only a few cases of narrow IAC with duplication have been reported to date, with the first case in 1997 by Casselman et al. [8] and the second case reported in 1999 by Vilain et al. of narrow IAC with a duplicated canal joined at the fundus of the IAC [10]. A third case was reported in 2000 by Cho et al. [11], and a fourth case was reported in 2003 by Ferreira et al. [2], describing a narrow IAC associated with enlargement of the bilateral vestibule and lateral semicircular canal, as well as dysplastic cochleae bilaterally.

HRCT of temporal bone is considered a modality of choice because of its excellent details of temporal bone and inner ear structure [1]. The main drawback of CT is its limited role in visualizing neural components. Seven cases of congenital sensorineural hearing loss were reported by Casselman et al., where five cases had aplasia or hypoplasia of the vestibulocochlear nerve with a normal IAC as detected on MRI [8]. It is essential to assess the cochlear nerve in cases with a narrow IAC to select patients for cochlear implantation [3]. In patients with aplasia of the cochlear nerve, cochlear implantation may not be beneficial. Hence, MRI is necessary to be performed in patients with sensorineural hearing loss to assess neural structures in IAC and is now recommended to be performed along with HRCT to assess abnormalities causing sensorineural hearing loss.

A detailed anatomical delineation can be attained by gradient echo sequences. Other sequences like 3D magnetization prepared rapid gradient echo (MP-RAGE), 3D balanced fast field echo (B-FFE), 3D Fourier transformation-constructive interference in the steady state (3D FT-CISS), and 3D DRIVE sequences are 3D submillimetric in spatial resolution for adequate evaluation of neural structures [11,12]. In our study, 3D constructive interference in the steady state sequence using a 1.5-Tesla MRI scanner was obtained to assess neural structures of IAC.

Wang et al. [15] reported 13 cases of narrow duplicated IAC with vestibulocochlear nerve usually appearing hypoplastic, with most of them being unsuitable for cochlear implantation. In this study, sensorineural hearing loss was the most common presentation with varying degrees of dysfunction since childhood, and facial nerve dysfunction was rare. All the patients had hearing dysfunction; two patients had vestibular dysfunction, and only one patient had facial nerve dysfunction. A rare case of a thick bony wall separating the middle from the inner ear with round and oval window atresia was documented. They concluded that duplicated IAC is a rare anomaly accounting for about 0.019% of patients with sensorineural hearing loss.

## Conclusions

A narrow IAC with duplication is a rare disorder with double-canal appearance being the characteristic feature on HRCT scans. Commonly, they are associated with vestibulocochlear nerve aplasia and middle or inner ear anomalies, with facial nerve dysfunction being an extremely rare entity. A complete past medical history and physical examination, including cranial nerve assessment, audiometry, HRCT, and MRI imaging, should be done in patients presenting with congenital sensorineural hearing loss.

## References

[1] Yates JA, Patel PC, Millman B, Gibson WS (1997). Isolated congenital internal auditory canal atresia with normal facial nerve function. Int J Pediatr Otorhinolaryngol.

[2] Ferreira T, Shayestehfar B, Lufkin R (2003). Narrow, duplicated internal auditory canal. Neuroradiology.

[3] Winslow CP, Lepore ML (1997). Bilateral argenesis of lateral semicircular canals with hypoplasia of the internal auditory canal (IAC). Arch Otolaryngol Head Neck Surg.

[4] Jackler RK, Luxford WM, House WF (1987). Congenital malformations of the inner ear: a classification based on embryogenesis. Laryngoscope.

[5] Rothschild MA, Wackym PA, Silvers AR, Som PM (1999). Isolated primary unilateral stenosis of the internal auditory canal. Int J Pediatr Otorhinolaryngol.

[6] Valvassori GE, Pierce RH (1964). The normal internal auditory canal. AmJ Roentgenol.

[7] Standring S (2008). Gray's anatomy: the anatomical basis of clinical practice.

[8] Casselman JW, Offeciers FE, Govaerts PJ, Kuhweide R, Geldof H, Somers T, D'Hont G (1997). Aplasia and hypoplasia of the vestibulocochlear nerve: diagnosis with MR imaging. Radiology.

[9] Ramírez-Camacho R, Berrocal JR, Arellano B (2001). Bilateral malformation of the internal auditory canal: Atresia and contralateral transverse megacrest. Otolaryngol Head Neck Surg.

[10] Vilain J, Pigeolet Y, Casselman JW (1999). Narrow and vacant internal auditory canal. Acta Otorhinolaryngol Belg.

[11] Cho YS, Na DG, Jung JY, Hong SH (2000). Narrow internal auditory canal syndrome: parasaggital reconstruction. J Laryngol Otol.

[12] Demir OI, Cakmakci H, Erdag TK, Men S (2005). Narrow duplicated internal auditory canal: radiological findings and review of the literature. Pediatr Radiol.

[13] Desai NK, Young L, Miranda MA, Kutz JW Jr, Roland PS, Booth TN (2011). Pontine tegmental cap dysplasia: the neurotologic perspective. Otolaryngol Head Neck Surg.

[14] Nixon JN, Dempsey JC, Doherty D, Ishak GE (2016). Temporal bone and cranial nerve findings in pontine tegmental cap dysplasia. Neuroradiology.

[15] Wang L, Zhang L, Li X, Guo X (2019). Duplicated internal auditory canal: high-resolution CT and MRI findings. Korean J Radiol.

